# Design and methodology of the impact of HemoDiaFIlTration on physical activity and self-reported outcomes: a randomized controlled trial (HDFIT trial) in Brazil

**DOI:** 10.1186/s12882-019-1247-8

**Published:** 2019-03-20

**Authors:** Roberto Pecoits-Filho, John W. Larkin, Carlos Eduardo Poli-de-Figueiredo, Américo Lourenço Cuvello Neto, Ana Beatriz Barra, Sinaia Canhada, Ludimila Guedim de Campos, Juliane Woehl, Priscila Bezerra Gonçalves, Hao Han, Thyago Proença de Moraes, Jochen G. Raimann, Maria Eugenia F. Canziani, on behalf of the HDFIT Study Investigators, Ana Claudia Dambiski, Thaylane Amanda de Souza, Daniela Ponce, Edwa Maria Bucuvic, Luciana Menin Ferreira, Wanderson de Souza Carvalho, Jorge Paulo Strogoff de Matos, Esther Oliveria Silva, Manuel Carlos Martins de Castro, Celina de Fátima e Silva, Maria Eugenia F Canziani, Silvia R Manfredi, Katia Santos, Ana Paula Fonseca Correia, Giovani Gadonski, Adriana Conti, Inah Pecly, Camille Souza Paixão, Viviane Calice-Silva, Simone Ribeiro, Lizia Regina Ribeiro Caldeira, Adailto Santos, Rosilene Motta Elias, Andreia Barbosa Dos Santos, Américo Lourenço Cuvello-Neto, Amanda Monteiro Virolli

**Affiliations:** 1 School of Medicine, Pontifícia Universidade Católica do Paraná, Imaculada Conceição 1155, Curitiba, PR 80215-901 Brazil; 2 Fresenius Medical Care North America, 920 Winter Street, Waltham, MA 02451 USA; 3 Pontifícia Universidade Católica do Rio Grande do Sul, Av. Ipiranga 6681, Partenon, Porto Alegre, RS 90619-900 Brazil; 4 Hospital Alemão Oswaldo Cruz, R. Treze de Maio 1815, São Paulo, São Paulo, 01327-001 Brazil; 5 Fresenius Medical Care Brazil, R. Amoreira 891, Jaguariúna, São Paulo, 13820-000 Brazil; 6 Health Technology Graduate Program, Pontifícia Universidade Católica do Paraná, Imaculada Conceição, 1155, Curitiba, PR 80215-901 Brazil; 7 Research Division, Renal Research Institute, 315 East 62nd Street, 4th Floor, New York, NY 10065 USA; 8 Universidade Federal de São Paulo, R. Sena Madureira 1500, São Paulo, São Paulo 04021-001 Brazil

**Keywords:** End stage renal disease (ESRD), Hemodialysis (HD), Hemodiafiltration (HDF), Physical activity, Dialysis recovery, Activities of daily living (ADL), Steps per day, Accelerometer

## Abstract

**Background:**

End stage renal disease (ESRD) patients require a renal replacement therapy (RRT) to filter accumulated toxins and remove excess water, which are associated with impaired physical function. Hemodialysis (HD) removes middle-molecular weight (MMW) toxins less efficiently compared to hemodiafiltration (HDF); we hypothesized HDF may improve physical function. We detailed the design and methodology of the HDFIT protocol that is testing whether changing from HD to HDF effects physical activity levels and various outcomes.

**Methods:**

HDFIT is a prospective, multi-center, unblinded, randomized control trial (RCT) investigating the impact of dialysis modality (HDF verses HD) on objectively measured physical activity levels, self-reported quality of life, and clinical/non-clinical outcomes. Clinically stable patients with HD vintage of 3 to 24 months without any severe limitation ambulation were recruited from sites throughout southern Brazil. Eligible patients were randomized in a 1:1 ratio to either: 1) be treated with high volume online HDF for 6 months, or 2) continue being treated with high-flux HD. This study includes run-in and randomization visits (baseline), 3- and 6-month study visits during the interventional period, and a 12-month observational follow up. The primary outcome is the difference in the change in steps per 24 h on dialysis days from baseline to the 6-month follow up in patients treated with HDF versus HD. Physical activity is being measured over one week at study visits with the ActiGraph (www.actigraphcorp.com). For assessment of peridialytic differences during the dialysis recovery period, we will analyze granular physical activity levels based on the initiation time of HD on dialysis days, or concurrent times on non-dialysis days and the long interdialytic day.

**Discussion:**

In this manuscript, we provide detailed information about the HDFIT study design and methodology. This trial will provide novel insights into peridialytic profiles of physical activity and various self-reported, clinical and laboratory outcomes in ESRD patients treated by high volume online HDF versus high-flux HD. Ultimately, this investigation will elucidate whether HDF is associated with patients having better vitality and quality of life, and less negative outcomes as compared to HD.

**Trial registration:**

Registered on ClinicalTrials.gov on 20 April 2016 (NCT02787161).

**Electronic supplementary material:**

The online version of this article (10.1186/s12882-019-1247-8) contains supplementary material, which is available to authorized users.

## Background

Patients with chronic kidney disease (CKD) often advance to end stage renal disease (ESRD) progressively developing uremia and requiring renal replacement therapy (RRT) to counteract symptoms and to sustain life. Hemodialysis (HD) is the most common RRT and typically includes intermittent, thrice-weekly treatments to remove toxins and excess fluid by means of ultrafiltration. Despite technological progress and advances in care paradigms for the management of ESRD, the incidence of adverse complications such as hospitalization and death remain notably high in this population [1]. Mortality rates for patients diagnosed with ESRD treated by HD are higher than patients diagnosed with cardiovascular diseases (CVDs), diabetes, or cancer alone [1]. Patients with ESRD commonly have low levels of vitality, a diminished quality of life, and poor nutritional status, conditions which associate with worse clinical outcomes in this population [2–5].

Several middle-molecular weight (MMW) molecules (e.g. beta-2 microglobulin, cytokines) have been identified as important uremic toxins, and their accumulation in those with decreased or lacking renal function has been associated poor clinical outcomes [6, 7]. Conventional HD has been reported to be limited in terms of MMW toxins removal [8]. Hemodiafiltration (HDF) is a modality that is assumed to have an enhanced removal of various circulating retention solutes through increased convective removal, which is due to higher volume removal and administration of substitution volume for the removed volume. However, there are inconsistent findings on the effects of HDF in terms of health-related quality of life (HRQOL) after dialysis treatments in the literature [9–14].

Physical activity and pervasive sensing continue to be of growing interest for the medical community and while associations to outcomes are established, intensive research efforts are underway to study specific populations and to what extent interventions promoting physical activity translate into improved outcomes [15–17]. Data from a recently conducted randomized control trial (RCT) indicated higher self-reported physical activity levels in patients receiving HDF compared to high flux HD [13]. Profiles of physical activity measured objectively by pedometers and accelerometers have been investigated in people with ESRD treated by the dialysis modalities of HD, peritoneal dialysis, and kidney transplant; physical activity levels qualitatively vary between patient treated with HD versus peritoneal dialysis, and quantitively have been shown to differ between patients treated with HD versus a kidney transplant [18–29]. Longitudinal data on objectively measured physical activity levels is scare in the HD population; the studies that have been conducted generally observed decreases with longer HD vintages that were reductions of more than 130 steps per day each year on HD [22, 28]. While the profiles of objectively measured physical activity levels are not well studied in the HD population [23, 24], to the best of the authors’ knowledge they have never been characterized in patients treated receiving HDF and never been compared to the HD population.

Herein we detail the study design and methodology of the *Impact of HemoDiaFIlTration on Physical Activity and Self-Reported Outcomes: A Randomized Controlled Trial* (HDFIT) conducted in Brazil that aims to characterize objectively measured physical activity levels and various self-reported and clinical outcomes in ESRD patients randomized to be treated with either high volume online HDF or high-flux HD. One novel attribute of this study is that it is capturing granular slices of physical activity levels in reference to the time of chronic dialysis treatments to investigate whether peridialytic differences exist, particularly in the post-dialysis period.

## Methods

### Objectives

We aim to evaluate the profiles and longitudinal patterns of objectively measured physical activity levels and other relevant outcomes (HRQOL, laboratories, medication use, intradialytic events, hospitalization events, and deaths) in ESRD patients randomized to be treated with either high volume online HDF or high-flux HD over for 6 months. We also aim to investigate selected outcomes (HRQOL, laboratories, deaths) during a post-interventional follow up 12 months after randomization.

### Primary hypothesis

1. High volume online HDF associates with higher physical activity levels captured by a triaxial accelerometer, as compared to high-flux HD.

### Secondary hypothesis

2. High volume online HDF associates with improved HRQOL outcomes (KDQOL scores, self-reported dialysis recovery time), as compared to high-flux HD.

### Exploratory hypotheses

3. High volume online HDF associates with better body hydration status (e.g. interdialytic weight gain, extracellular water volume, dry weight), as compared to high-flux HD.
4. High volume online HDF associates with improved thirst questionnaire scores, as compared to high-flux HD.
5. High volume online HDF associates with better laboratory outcomes (e.g. hemoglobin, transferrin saturation, MMW clearance), as compared to high-flux HD.
6. High volume online HDF associates with distinct patterns of medication use, particularly in terms of need for erythropoietin stimulating agents, phosphate binders, and anti-hypertensive medication, as compared to high-flux HD.
7. High volume online HDF associates with lower intradialytic morbid event rates (e.g. intradialytic hypotension, nausea, muscle cramps), as compared to high-flux HD.
8. High volume online HDF will be associated with lower rates of all-cause hospital admissions and length of stay, as compared to high-flux HD.
9. High volume online HDF will be associated with decreased all-cause mortality rates, as compared to high-flux HD.

### General study design

**Fig. 1:**
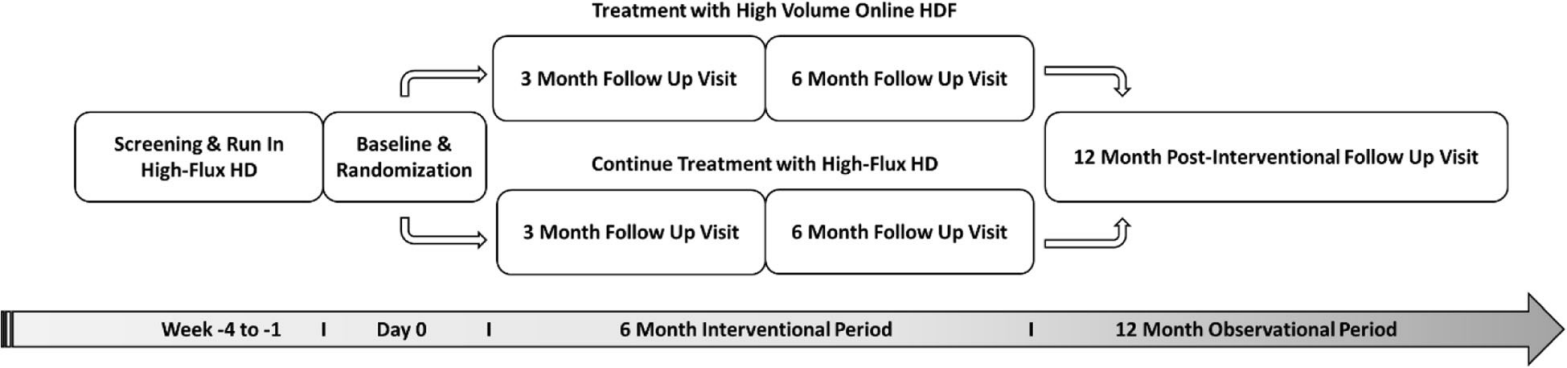
Schematic of the HDFIT Study Design

HDFIT was designed as a prospective, multi-center, unblinded, randomized control trial (RCT) studying the impact of dialysis modality (high volume online HDF versus high-flux HD) on objectively measured physical activity levels and clinical outcomes. We recruited adult ESRD patients, who received standard-of-care HD in an outpatient dialysis center in the south and southeastern regions of Brazil, who initiated RRT at least 3 months, but no more than 24 months prior to time of randomization. Patients were recruited by investigators at each site and those who provided written consent underwent a screening and run-in period of up to 4 weeks. During these 4 weeks patients received high-flux HD for the run-in period during which baseline assessments for demographic, clinical, social, and physical activity parameters were performed. Patients who were treated with low-flux HD as the standard-of-care and were converted to high-flux HD for the run-in period. Those who successfully completed the run-in period and continued to meet study eligibility criteria were randomized in a 1:1 ratio, stratified by study site and dialysis shift, to either be treated by high volume online HDF or continue to receive high-flux HD for a 6-month period. At the randomization visit, baseline values for laboratory and quality of life parameters were collected. Two study related visits were scheduled during the follow up period: one at 3 and one at 6 months after randomization with similar assessments of clinical, laboratory, and physical activity parameters (Table 1). Between study visits and procedures, patients receive standard-of-care with all prescriptions at the discretion of the treating nephrologist and care teams in the clinics. Clinical, laboratory, and treatment related patient data from the provision of standard-of-care dialysis is collected 12 months after randomization. Figure 1 schematically depicts the study design.

**Table 1:** Schedule of Events and Data Collection

Study Event	Screening	Enrollment	3-Month	6-Month	12-Month
Informed Consent	X				
Run In	X				
Randomization and Allocation		X			
Intervention (HDF or HD)		X	X	X	
Assessments:					
*Demographics*	X	X			
*Clinical and Treatment Parameters*	X	X	X	X	X
*Outcome Variables*					
*Physical Activity Levels*	X		X	X	
*HRQOL*		X	X	X	X
*Other data variables*					
*Laboratory Parameters*		X	X	X	X
*Biorepository Specimens*		X	X	X

### Ethical considerations

The HDFIT RCT (clinicaltrials.gov registry # NCT02787161) was designed and is conducted in accordance with the Declaration of Helsinki under a protocol approved by the Pontifícia Universidade Católica do Paraná ethics review board (ERB) (central application # 54926916.7.1001.0020; approval number 1.538.784). All patients provided written informed consent in Portuguese for their participation in this protocol before any study procedures were performed. Participants’ protected health information was deidentified by the investigators to ensure confidentiality. Study status reports are actively being performed on the registry and communicated to the ERB, centers, investigators and patients.

### Setting

We invited the governing bodies of 14 outpatient dialysis centers in the south and southeastern regions of Brazil to participate as sites for recruitment of study participants. Investigators were permitted to recruit up to 20 patients per study site; after achieving recruitment goals, sites were considered for further recruitment of participants upon formal request to the steering committee. This study is being coordinated and managed by the EPICENTER academic clinical research organization (ACRO) based at Pontifícia Universidade Católica do Paraná.

### Characteristics of participants

The treating nephrologist and associates served as the local investigators in the clinics who aided the identification, recruitment, and evaluation of potential study candidates for the trial based on the following inclusion and exclusion criteria:

#### Inclusion criteria

1. Female or male ESRD patient treated with standard high- or low-flux HD thrice-weekly for at least 3 months and no more than 24 months.
2. Clinically stable patients per the medical judgment and clinical evaluation of the investigator.
3. Patients who are adequately dialyzed achieving a minimal dialysis dose Kt/V ≥ 1.2.
4. Patients who are dialyzed utilizing an arteriovenous fistula/graft or permanent central-venous catheter as the dialysis access. Access was required to have adequate flow as per the medical judgment and clinical evaluation of the investigator.

#### Exclusion criteria

1. Patients younger than 18 years.
2. Patients that are determined by the investigator to have life expectancy less than 3 months because of non-ESRD related comorbidities.
3. Patients currently participating in another clinical research trial.
4. Patients who are considered non-adherent with the frequency and/or duration of their prescribed HD treatment by the investigator.
5. Patients with a severe limitation(s) in their mobility/ambulation (e.g. patients suffering from an amputation, neurologic disorder, or muscular disorder that seriously affects their activities of daily living) per the medical judgment of the investigator.

### Protocol addendums

The protocol, ICF and study documents were approved by the ERB before the commencement of any study activities. The original protocol was approved by the ERB on 11 May 2016. The ICF, study questionnaires, and diaries were approved 29 Jun 2016. After the initiation of the trial, there were multiple minor addendums/changes made to the protocol and/or study documents. This included the revision of typographical error in a questionnaire that was approved on 29 Sep 2016. There was a protocol addendum that included clerical updates to more comprehensively describe various study procedures and more clearly detail the capture of routine standard of care treatment data during participation in the trial, which was approved by the ERB on 05 Apr 2017. Another addendum to the protocol to allow for the addition investigative study sites to recruit participants was approved by the ERB on 07 Jun 2017. Furthermore, there was a protocol addendum to the eligibility criteria that extend the permitted inclusionary dialysis vintage from 18 to 24 months, clarify that the dialysis access of arteriovenous grafts was inclusionary along with fistulas and permanent central-venous catheters, and more clearly define exclusionary severe limitation(s) in mobility/ambulation, which was approved by the ERB on 21 Mar 2018. The last protocol addendum included an addition of a 12-month follow up after randomization for collection of patient data recorded in the provision of routine dialysis; this was approved by the ERB on 04 Apr 2018.

### Study visit procedures

#### Screening and run-in

Patients who were potential candidates for the HDFIT trial were invited by the investigators to be screened for eligibility; those confirmed to be eligible were approached for recruitment. The investigator reviewed the informed consent form (ICF), study procedures, risks, and benefits with each patient. In addition to study procedures, the ICF also included the details on the collection of biorepository specimens for use in ancillary studies of biomarkers (e.g. MMW toxins). Interested patients provided their signed consent on two copies of the ICF; both ICFs were signed and executed by the investigator. One of the two executed ICFs was filed in the patient’s medical record and the other was given to the patient for their records.

After signing the ICF, participants entered the screening and run in period for a period for up to 4 weeks. During the screening and run-in period patients receiving high-flux HD as a standard of care continued to undergo high-flux HD and could have baseline assessments captured and move onto enrollment. Patients using low-flux HD as a standard of care were switched to high-flux HD for the full 4-week run in period. All HD treatments during the screening and run in period were performed using standardized high-flux dialyzers. Baseline physical activity levels were objectively measured over 7 days during the screening and run-in period. Baseline demographic, clinical, and social data were recorded from the patient’s standard of care medical records, the investigator assessments, and/or per patient report. Baseline parameters recorded in the screening and run-in period included: demographics (age, sex, race) social parameters (educational level, family income level, employment status, transportation method to clinic, distance from the clinic to the patient’s residence), dialysis access type (arteriovenous fistula, graft or permanent catheter), clinical characteristics (height, weight), and treatment characteristics. The investigators reviewed the patients’ medical history, clinical case, and laboratory data during the screening and run-in period; those identified by the investigator to meet all inclusion and no exclusion criteria were scheduled to proceed to be randomized into the trial.

#### Randomization

On the day of the randomization visit, patients were confirmed to meet eligibility criteria, enrolled into the trial, and randomly allocated to either receive high volume online HDF or continue high-flux HD for 6 months and be observed for the next 12 months. Sequence generation for the randomized allocation was performed in a centralized manner by the EPICENTER ACRO using Microsoft Excel; the randomization allocation was uploaded into REDCap for assignment. The investigators’ staff performed the randomization via the REDCap™ (https://www.project-redcap.org) electronic case report form (eCRF) and assigned patients to either arm. Because of the nature of the intervention, the investigators and local clinical staff could not be blinded to treatment assignment.

Before initiation of the interventional treatments (high volume online HDF or high-flux HD), baseline values for clinical, laboratory, and quality of life parameters were collected at the randomization visit. These included capturing clinical parameters (weight, systolic and diastolic blood pressures (SBP/DBP), and pulse), performance of a bioelectrical impedance body composition assessment using the Body Composition Monitor (BCM; Fresenius Medical Care, Bad Homburg, Germany) [30, 31] before dialysis, collection of blood samples, administration of the HRQOL questionnaires (KDQOL, self-reported dialysis recovery time, thirst questionnaire), recording the participants medical history (including presence of comorbidities and etiology of kidney disease), concomitant medications, and dialysis treatment characteristics (treatment time, ultrafiltration volume (UFV), substitution volume for HDF, urea distribution volume, estimated adequacy (single pool Kt/V), blood flow rate (Qb)). After initiation of the interventional treatments, adverse events were actively assessed by the investigators, and safety data was collected as applicable.

Laboratory parameters were captured from either routine standard-of-care laboratories analyzed at local or central locations and included: pre- and post-dialysis blood urea nitrogen (BUN), creatinine, hemoglobin, hematocrit, alanine aminotransferase (ALT), calcium, potassium, phosphorous, glucose (only in diabetics), intact parathyroid hormone (iPTH), alkaline phosphatase, serum iron, ferritin, transferrin saturation (TSAT), total protein, albumin, globulin, albumin/globulin ratio, hepatitis B surface antigen (HBsAg), hepatitis B surface antibody (anti-HBs), hepatitis C antibody (anti-HCV), and Vitamin D. Calculations were performed to determine the adequacy (single-pool Kt/V) and urea reduction ratio (URR) based off pre-and post-dialysis BUN levels. Additional pre- and post-dialysis biospecimens were collected and stored in a biorepository for future exploratory analyses.

#### Follow up

During the follow up period, patients are treated for 6 months with high volume online HDF or high-flux HD and observed for 12 months after randomization. There are two study related follow up visits at 3- and 6-months after randomization and initiation of high volume online HDF or continued high-flux HD. These visits include consistent procedures with the randomization visit for the capture of physical activity levels over one week, capture of clinical parameters, performing BCM before dialysis, collection of study biospecimens, administration of the HRQOL questionnaires, assessment of changes in concomitant medications. Throughout the 6 month interventional period, dialysis access issues and episodes of intradialytic hypotension (health care provider reported and based on European Best Practice Guidelines [32]) were captured at every dialysis session and patients were assessed for adverse events. Additionally, routine laboratories and dialysis treatment characteristics from each treatment are collected from the patient’s standard-of-care medical records. In the post-interventional period, there is a 12-month follow up where routine standard-of-care clinical parameters, HRQOL questionnaire scores, laboratories, and events (e.g. transplants, modality changes, deaths) are collected from the patient’s medical records.

### Early withdraw/discontinuation

Patients can withdraw consent in the trial at anytime and will not have any further data collected. The investigators can withdraw patients from the trial or discontinue the interventional treatment in patients randomized to HDF. Patients who discontinue the interventional treatment will have their data captured and continue to perform all other study procedures, unless the patient or investigator withdraws the patients form the study.

### Study status and timeline

Fourteen study sites were activated and involved in the recruitment and screening of participants starting in July of 2016. The trial successfully enrolled 195 patients from 13 study sites by October of 2017. Follow-up study procedures are ongoing and study data will continue to be collected until approximately November of 2018. A description of the participant flow is depicted in Fig. 1.

### Study specific materials

#### Source documentation and case report forms

Study data is being collected on universal source documents that are recorded in the REDCap™ (https://www.project-redcap.org) eCRF by the investigators’ and their designated coordinators. The eCRF was designed and created through the REDCap platform by the EPICENTER study team at Pontifícia Universidade Católica do Paraná. Study data is being managed using REDCap electronic data capture tools hosted at Pontifícia Universidade Católica do Paraná [33, 34]. REDCap (Research Electronic Data Capture) is a secure, web-based application designed to support data capture for research studies, providing 1) an intuitive interface for validated data entry; 2) audit trails for tracking data manipulation and export procedures; 3) automated export procedures for seamless data downloads to common statistical packages; and 4) procedures for importing data from external sources. The eCRF software is validated and compliant with international privacy and compliance standards for RCTs (US 21 CFR part 11, FISMA (low, moderate, high), EU Annex 11). All data is being collected in an anonymized manner consistent with ERB and International Conference on Harmonisation (ICH) guidelines.

#### Trial monitoring

Site level trial monitoring is being performed for protocol adherence and source data verification to the eCRFs. Additionally, central clinical research associates at the EPICENTER are performing centralized monitoring of the eCRFs. Serious adverse events are actively reviewed and monitored by the steering committee comprised of nephrologists. A formal Data and Safety Monitoring Board was not appointed for this study due to use of an approved device as the intervention. Any issues pertaining to nonadherence with eligibility of a randomized participant, allocation of interventions, or concerns relating to adverse events are being discussed with and reviewed by steering committee.

#### Dialysis, machines, and equipment

Each study site was provided with two dialysis machines capable of performing high volume online HDF (Fresenius 5008 CorDiax®). Sites are routinely supplied with dialyzers (Fresenius Polysulfone HDF 100®), bloodlines (Fresenius LifeLine_Beta_® adult bloodlines), and concentrates (Fresenius bibag® dry bicarbonate concentrate and liquid acid concentrate) for performing 6 months of HDF treatments for participants that were randomized to the high volume online HDF group. High-flux HD was performed using Fresenius 4008S machines, bloodlines, and concentrates used at the dialysis clinic sites for the run-in period and for patients randomized to the high-flux HD group; high-flux dialyzers (Fresenius FX Classix 100 (HD arm) and Fresenius Polysulfone HDF 100 (HDF arm)) were provided to the study sites for all study related treatments.

A Body Composition Monitor (Fresenius Medical Care, Bad Homburg, Germany) was supplied to the centers that did not previously have one to accurately monitor fluid status, measure body composition, and to determine the patients’ optimal post-dialysis weight for dialysis treatments performed in this trial. Both BCM and dialysis treatment data was captured in source documents and eCRF.

Investigators and clinical professionals at each participating center were trained by the study team in a standardized fashion to ensure there was homogeneity in dialysis treatment and study procedure. Routine study meetings are being performed for continuing training. This protocol recommends a standardized dialysis protocol following the current guidelines both for high flux HD or high volume online HDF. Postdilution high volume online HDF is being performed using the Fresenius 5008 CorDiax HDF machine with the AutoSub plus function and high-flux HD is being performed using the Fresenius 4008S machine. The recommended composition of the substitution fluids and dialysate was: sodium 138 mEq/L, potassium 2 mEq/L, calcium 1.5 mmol/L, dry bicarbonate 32 mEq/L and glucose 5.5 mmol/L. Dialysis needle size was standardized for high volume online HDF. For high volume online HDF, sterile and nonpyrogenic substitution fluids were produced by ultrafiltration of the ultrapure dialysate. The HD group also used ultrapure dialysate. Ultrapure quality was defined as bacterial counts < 0.1 CFU/mL and endotoxin levels < 0.025 EU/mL. Fresenius Polysulfone HDF 100 dialyzers are being used for high volume online HDF treatments and Fresenius FX Classix 100 dialyzers are being used for HD treatments. The intended dialysis treatment duration for both modality arms of the trial was 240 min. For the high volume online HDF arm, dialysis is being performed in the post-dilution mode with a target convection volume of 22 L/treatment.

#### Physical activity monitoring device

We are objectively measuring the patients’ physical activity levels over 7 days at the run-in and the 3- and 6-month follow up visits using a validated tri-axial accelerometer (ActiGraph™ wGT3X-BT model; http://www.actigraphcorp.com) that measures detailed parameters of physical activity [23, 35–38]. The ActiGraph™ is lightweight (19 g), worn at the waist, interfaces with the internet via Bluetooth, and date- and time-stamped activity levels are being uploaded at routine dialysis visits. Activity during sleep cannot be accurately determined with use of a waist worn physical activity monitor, therefore we ask patients to take off the physical activity monitor during sleep. Additionally, the physical activity monitor is being removed during bathing.

#### Diary logs

During the 7 days of physical activity monitoring patients are completing a diary that records the time of their dialysis treatment, sleep, bathing, and any time that they are not wearing the physical activity monitor. The physical activity monitoring has/is being started before the dialysis treatment in the run-in period, at the 3- and 6-month visit. Each patient is given a paper diary and instructed how to complete the diary by the investigator or their designee. On the first day of activity monitoring, the patients were given a diary with their study patient number denoted on it and they record their age, weight, and height; the investigative staff assisted patients in completion of their information as needed. Throughout the 7 days of activity monitoring, patients recorded the start and end time of dialysis (on dialysis days), the time they go to sleep and wake, the time they start and end bathing, and any time that they did not wear the physical activity monitor for more than 30 min.

#### HRQOL questionnaires

HRQOL is being assessed at the baseline and follow-up study visits with the Kidney Disease Quality of Life - Short Form (KDQOL-SF) version 1.3 that has been adapted and validated to Brazilian Portuguese [39]. The KDQOL-SF consists eight domains that can be summarized in two summary scores, one for physical functioning (the physical composite score (PCS)) and one for mental functioning (the mental composite score (MCS)). After initiation of the study, it was identified that there was one question in the KDQOL-SF survey that had a typographical error in up to 100 surveys; this question was supposed to read as follows: “do you get sick a little easier than other people?”. However, the typographical error made the question ask patients “do you get obey a little easier than other people?”. After identification of the error in this survey, study coordinators adjudicated and corrected the patients’ response to the question answer if it was incorrect.

In addition, HRQOL is being assessed by the Time to Recover from Dialysis Question, “How long does it take you to recover from a dialysis session?”; patients could answer a time in minutes after dialysis. This question has been shown to have both convergent and divergent validity in HD patients, and demonstrated to be sensitive to change [40]. Moreover, participants will also have HRQOL assessed via Dialysis Thirst Inventory, which is a seven-item questionnaire that five point Likert type scale for each item (never = 1, almost never = 2, sometimes = 3, often, very often = 5) [41]. The Dialysis Thirst Inventory has been shown in an RCT to be positively correlated to interdialytic weight gain [42], and found to have consistent detection of thirst/dry mouth as the validated Xerostomia Inventory (XI) [41]. All questionnaires will be administered to patients by staff who will help the patients to fill out the forms, if necessary.

#### Biomarker laboratory specimens

Pre- and post-dialysis study specific blood specimens were collected at the randomization visit and will be collected at the 3- and 6-month follow up visits for biorepository and exploratory analysis of biomarkers of inflammation, oxidative stress, and uremic toxins. These include the collection of 8–10 mL of blood in two different tubes (serum and plasma), processing and storage at the temperature of − 20 C by the clinic team. All biomarker specimens are transferred to the central lab and stored at − 80 C (PUCPR Multiuser Core Facility: Imaculada Conceição Street, 1155 - Zip Code: 80215–901 Curitiba, Paraná, Brazil).

### Power analysis

Data will be analyzed according to an intention-to-treat principle (i.e. according to assigned instead of received treatment). The primary endpoint is steps per 24 h beginning at the start of each dialysis treatment on dialysis days and concurrent times on non-dialysis days in patients treated with high volume online HDF versus high-flux HD. With an enrollment target of 110 participants in each arm (considering a drop out of 20%) we estimated the 86 patients will complete the follow up in each arm, which will provide the trial a 90% power to detect a 20% effect with respect to the primary outcome (predefined as a 20% increase in average total steps at the dialysis day in the high volume online HDF group compared to the high flux HD group).

In addition, near the end of the baseline period we confirmed the power calculation using interim baseline data (*n* = 142); this computation suggested that enrollment of 120 subjects would be anticipated to detect a 10% effect with respect to the primary outcome with 90% statistical power, and with 90 subjects it would be anticipated to detect an effect with respect to the primary outcome with 80% statistical power.

### Physical activity analysis

Accelerometry continuously records data over 7 days of activity monitoring. Physical activity data in this study is being extracted from the accelerometer files for times relative to the beginning of dialysis to 24 h after the initiation of dialysis, and concurrent/parallel times to dialysis on the following first non-dialysis days and the second long interdialytic non-dialysis day. We are extracting data from the physical activity monitor using the ActiLife v6.13.3 software (http://actigraphcorp.com/support/software/actilife/). Raw physical activity data is uncompressed and bandpass filtered using the default filter with an epoch length of 60 s. The validation and filtering non-wear time readings is being performed using vector magnitude and the Choi 2011 algorithm [43], with the custom setting of 60 min for the window of consecutive zero or non-zero measurements [44]. Notably, we did test the utility of the low frequency filter available with the ActiGraph activity monitor yet identified overcounting with discrepancies of more than 2000 steps per hour in some instances compared to the default filter.

The physical activity levels are cleaned, sliced, computed and exported from the ActiLife software using a custom coded file to denote predefined granular slices based off the start and end time of each dialysis session for each patient, as well as concurrent/parallel times to dialysis on the following first non-dialysis days and the second long interdialytic non-dialysis day. For the computation physical activity levels, we are using the Freedson VM3 Combination (2011) algorithm to calculate energy expenditure and most moderate to vigorous physical activity (MVPA) level cut points [45], as well as, the Freedson Adult 1998 algorithm to calculate the metabolic rates (MET) [46]. The cut points used for physical activity levels of MVPA are as follows: Sedentary = 0–99 counts per minute (CPM); Light = 100–2689 CPM; Moderate Activity = 2690–6166 CPM; Vigorous = 6167–9642 CPM; Very Vigorous = 9643-infinity CPM. We defined bouts of physical activity being as 10 consecutive minute periods of MVPA and sedentary bouts as 10 consecutive minute periods of no physical activity. In instances that patients do not adhere to the protocol and wore the physical activity monitor during sleep, we code the export files to exclude this protocol defined non-wear time from the activity data using the sleep times reported by the patient in their diary log.

**Fig. 2:**
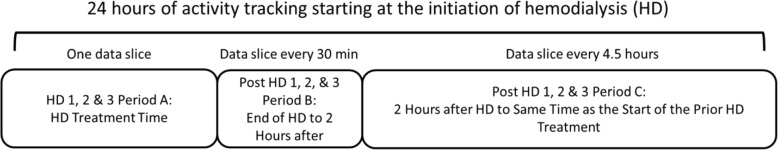
Schematic of Slicing of Physical Activity Data on Dialysis Days

**Fig. 3:**
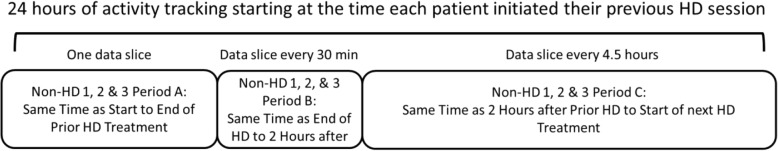
Schematic of Slicing of Physical Activity Data on Non-Dialysis Days

**Fig. 4:**
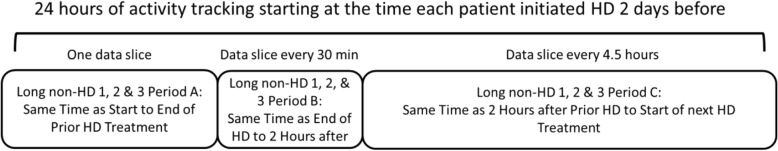
Schematic of Slicing of Physical Activity Data on Long Interdialytic Day

To allow for the assessment of granular physical activity levels relative to the timing of dialysis, the data is being extracted from the accelerometer files in predefined periods in 3 blocks (A, B, C) consisting of a total of 9 slices for each 24-h period on dialysis days (0 to ≤24 h after dialysis start; Fig. 2), first non-dialysis days (> 24 to ≤48 h after dialysis start; Fig. 3), and the second long interdialytic non-dialysis day (> 48 to ≤72 h after dialysis start; Fig. 4).

The extraction of data in Block A captures per patient physical activity data during dialysis (approximately 0 to ≤4 h after dialysis start), or data from concurrent/parallel times to dialysis on the subsequent first non-dialysis days (> 24 to ≤28 h after dialysis start) and second long non-dialysis day (> 48 to ≤52 h after dialysis start). Block A contains one slice of data.

The extraction of data in Block B captures per patient physical activity data starting the minute after completion of dialysis to 2 h post-dialysis (> 4 to ≤6 h after dialysis start), or data from concurrent/parallel times on the subsequent first non-dialysis days (> 28 to ≤30 h after dialysis start) and second long non-dialysis day (> 52 to ≤54 h after dialysis start). Block B has 4 predefined data slices of 30 min each in the 2-h period.

The extraction of data in Block C captures per patient physical activity data starting 2 h post-dialysis to 20 h post-dialysis (> 6 to ≤24 h after dialysis start), or data from concurrent/parallel times on the subsequent first non-dialysis days (> 30 to ≤48 h after dialysis start) and second long non-dialysis day (> 54 to ≤72 h after dialysis start). Block C has 4 predefined data slices of 4.5 h each in the 18-h period. The last 4.5-h slice in Block C may be truncated or extended to the start time the following dialysis session, if applicable.

### Statistical design

For the analysis of study data, we plan to perform descriptive calculation and tabulation of the average profiles of recruitment, demographics, clinical parameters, and physical activity levels. The distribution of physical activity levels (e.g. steps per 24 h and MVPA) will be computed and analyzed for normality using histograms and Kolmogorov Smirnoff normality test. Average values for all categorical variables will be calculated in counts or proportions. Continuous variables will be determined by calculating the mean with the standard deviation (SD) or median with 25th and 75th percentile. We plan to report physical activity levels by the 24-h post-dialysis period on dialysis days, as well as on concurrent periods on non-dialysis days and the long interdialytic day.

In subgroup analyses, we plan to establish granular profiles of physical activity levels after dialysis treatments, which will include analyses that explore if there are any differences between the dialysis days, non-dialysis days and the long interdialytic day, as well as differences between the first, second and third dialysis session of the calendar week. We plan to perform additional subgroup and exploratory analysis to investigate if differing demographic, clinical, laboratory, treatment, and other parameters influence physical activity levels. Further analysis may include additional profiles of energy expenditure (e.g. kilocalories, metabolic equivalent of task (MET) rates), body position (e.g. sitting, standing, walking), and other metrics available from the activity monitor.

We plan to perform an explorative analysis of missing treatments and missing data to evaluate if missing completely at random (MCAR), missing at random (MAR) or missing not at random (MNAR). Complete case analysis and multiple imputation will be considered based on frequency of missing data and nature of missing patterns.

The hypothesis driven outcomes of this study include the:

1. Primary outcome: the difference in the change in steps per 24 h on dialysis days (captured by a triaxial accelerometer and in reference to the start time of the dialysis) from baseline to the 6-month follow up in patients treated with high volume online HDF versus high-flux HD.
2. Secondary outcome: the difference in the change in KDQOL scores (total, PCS and MCS) and self-reported dialysis recovery time from baseline to the 6-month follow up in patients treated with high volume online HDF versus high-flux HD.
3. Various exploratory and subgroup outcomes will also be assessed to investigate hypotheses.

For the primary outcome of step counts per 24 h, we plan to use an intention-to-treat design to determine the effect of the intervention using linear mixed effects model comparing mean changes from baseline between treatment groups at months 3 and 6 with data presented in box-plots and histograms for the baseline, 3- and 6-month study periods. Similar methods will be used to assess exploratory HRQOL and other outcomes. Further evaluation of statistical interaction between the treatment effect on primary outcome may be performed for age, sex, body composition and other parameters.

An exploratory as-treated analysis may also be performed on the primary outcome, which will be restricted to those that achieved the target convection volume of 22 L per treatment at most dialysis sessions and did not miss a remarkable proportion of their dialysis treatments. This analysis will be performed using the same methods as the intention-to-treat analysis. Adjustments may be performed to the analysis via inverse probability weighting and g-estimation methods.

#### Key roles

The role of the study investigators was to assist with the design of the protocol and is to perform the medical oversight of the conduct of the study. Site investigators are directly performing and orchestrating the collection of study data at their sites during the trial. The principal investigator of the trial is providing medical oversight of the study conduct at all sites under the guidance of the steering committee and orchestrating the management of the study.

The role of the proponent institution Pontifícia Universidade Católica do Paraná is supporting the study with the infrastructure for the study management through use of the university’s academic clinical research organization, hosting of the REDCap eCRF on the university’s server, and use of the university’s central ERB and Research Council.

The role of the outpatient dialysis centers is permitting clinical research at the clinics and providing support of their clinical staff for the data collection and conduct of study procedures under the oversight of the site investigators and local trial leadership.

The role of the company Fresenius Medical Care is providing the sites with the infrastructure of HDF machines, providing the dialysis supplies during the study, as well as providing BCM machines as needed for the conduct of the study. Also, they are supporting the trial by providing some staff for monitoring of study sites. Furthermore, Fresenius Medical Care is providing a monetary award to PUCPR’s academic clinical research organization (EPICENTER) performing the central management, data acquisition, monitoring of the trial. Fresenius Medical Care and the subsidiary company Renal Research Institute are also providing support from statistical experts to assist in the analysis of trial data under the oversight of the steering committee. Moreover, Fresenius Medical Care has supported three investigator meetings, as well as three steering committee meetings. The leadership of Fresenius Medical Care reviewed and approved the protocol prior to commencement.

The role of the steering committee members that represent all supporting institutions is to review and approve the research design, protocol, addendums and changes to the protocol, analyses, and publications of study data, as well as provide oversight of the conduct and safety of the study.

## Discussion

We initiated a multi-center RCT that will characterize for the first time objectively measured physical activity levels in ESRD patients treated by high volume online HDF versus high-flux HD. One of the novel attributes of this study is that it will capture granular physical activity data in reference to the time of dialysis treatments. This approach will allow for the assessment of peridialytic differences during recovery from a chronic dialysis treatment. Furthermore, this study will define profiles of HRQOL in ESRD patients treated by the modalities of high volume online HDF versus high-flux HD and allow for the exploratory investigation of whether self-reported HRQOL is associated with physical activity levels. Other exploratory investigations will investigate the impact of modality and role of physical activity levels on various outcomes related to medication use, clinical characteristics, intradialytic events, hospitalizations, and deaths. Ultimately, this investigation will elucidate whether treatment of ESRD patients with high volume online HDF is associated with patients having higher vitality, a better quality of life, and less negative outcomes as compared to those treated with high-flux HD.

The design of this study has many strengths that include being a multicenter RCT with enrollment targets able to achieve the power needed to make sound comparisons for outcomes in a patient sample representative of the dialysis population in the south and southeastern regions of Brazil with a dialysis vintage less than two years. Of note, about 50% of the dialysis population in Brazil resides in the southern part of the country [47]. Furthermore, this is the first study to the knowledge of the authors to investigate granular physical activity levels based on the time of intensive treatments in a chronically diseased population. Moreover, the array of self-reported, clinical and laboratory outcomes makes this study unique in the ability to investigate various outcomes in patients treated with high volume online HDF or high-flux HD.

Although our study design has many strengths, there are some possible limitations that include a potential for variations with self-reported outcomes and a possibility of undercounting of physical activity levels due to the ActiLife software default algorithm that was validated in younger patients performing exercise [45, 46]. Our examination of the low-frequency-extension filter algorithm available with the ActiLife software identified evident overcounting, which is consistent with previous reports in elderly populations [48]. Nonetheless, physical activity will have internal consistency in this well controlled study. Another potential limitation of the trial is related to the unblinded study design, which could have potentially introduced bias depending on provider perceptions of HDF and the quality of the provider-patient relationship.

## Conclusions

We have thoroughly detailed the description of the HDFIT study design, methods and protocol. Ultimately, this RCT will characterize granular physical activity levels based on the timing of chronic dialysis treatments and various self-reported, clinical and laboratory outcomes in ESRD patients treated by high volume online HDF versus high-flux HD. The results of this study will provide novel insights into the impacts of routine dialysis therapies and modalities and characterize ESRD patient profiles during the recovery period after dialysis.

## Additional file

Additional file 1: Appendix A: HDFIT Study Site Investigators and Trial Leadership. List of the HDFIT study site investigators and the lead research coordinators. (PDF 123 kb)

## Abbreviations

ACRO: Academic clinical research organization
ADL: Activities of daily living
ALT: Alanine aminotransferase
Anti-HBs: Hepatitis B surface antibody
Anti-HCV: Hepatitis C antibody
BCM: Body composition monitor
BUN: Blood urea nitrogen
CKD: Chronic kidney disease
CPM: Counts per minute
CVD: Cardiovascular disease
DBP: Diastolic blood pressure
eCRF: Electronic case report form
ERB: Ethics review board
ESRD: End stage renal disease
HBsAg: Hepatitis B surface antigen
HD: Hemodialysis
HDF: Hemodiafiltration
HRQOL: Health related quality of life
ICF: Informed consent form
ICH: International Conference on Harmonisation
iPTH: intact parathyroid hormone
KDQOL: Kidney Disease Quality of Life
Kt/V: Dialysis adequacy
MAR: Missing at random
MCAR: Missing completely at random
MCS: Mental composite score
MET: Metabolic rates
MMW: Middle-molecular weight
MNAR: Missing not at random
MVPA: Moderate to vigorous physical activity
PCS: Physical composite score
Qb: Blood flow rate
RCT: Randomized control trial
RRT: Renal replacement therapy
SBP: Systolic blood pressure
TSAT: Transferrin saturation
UFV: Ultrafiltration volume
URR: Urea reduction ratio
