# Supplementary material for: Design and methodology of the impact of HemoDiaFIlTration on physical activity and self-reported outcomes: a randomized controlled trial (HDFIT trial) in Brazil
Source: BMC Nephrol. 2019 Mar 20;20:98. doi: 10.1186/s12882-019-1247-8 (PMC6425582; doi:10.1186/s12882-019-1247-8)
Supplement: Supplementary file 1 — Appendix A: HDFIT Study Site Investigators and Trial Leadership. List of the HDFIT study site investigators and the lead research coordinators. (PDF 123 kb) [file 12882_2019_1247_MOESM1_ESM.pdf]

Appendix A: HDFIT Study Site Investigators and Trial Leadership

Irmandade da Santa Casa de Misericórdia de Curitiba, Curitiba, Paraná

- Principal Investigator: Ana Claudia Dambiski, MD; anacdferraz@gmail.com
- Lead Coordinator: Thaylane Amanda de Souza, RN; thaylane@msn.com

Hospital de Clínicas - FMB - UNESP, Botucatu, São Paulo

- Principal Investigator: Daniela Ponce, MD; dponce@fmb.unesp.br
- Lead Coordinator: Edwa Maria Bucuvic, RN; embucuvic@fmb.unesp.br

Nefron Contagem, Contagem, Minas Gerais

- Principal Investigator: Luciana Menin Ferreira, MD; luciana.menin@nefron.com.br
- Lead Coordinator: Wanderson de Souza Carvalho, RN; wanderson.souza@nefron.com.br

Clínica de Diálise Ingá, Rio de Janeiro, Rio de Janeiro

- Principal Investigator: Jorge Paulo Strogoff de Matos, MD; strogoff@uol.com.br
- Lead Coordinator: Esther Oliveria Silva, RN; esodsilva@gmail.com

Instituto de Nefrologia de Taubaté, Taubaté, São Paulo

- Principal Investigator: Manuel Carlos Martins de Castro, MD; mmcastro@inefro.com.br
- Lead Coordinator: Celina de Fátima e Silva, RN; ivone_correa@inefro.com.br

Hospital do Rim e Universidade Federal de São Paulo, São Paulo, São Paulo

- Principal Investigator: Maria Eugenia F Canziani, MD; dialisefor@uol.com.br

- Lead Coordinator: Silvia R Manfredi, RN; silvia.hemod@hrim.com.br

Clínica de Doenças Renais – Botafogo, Rio de Janeiro, Rio de Janeiro

- Principal Investigator: Katia Santos, MD; katia.santos@grupocdrrj.com.br
- Lead Coordinator: Ana Paula Fonseca Correia, RN; ana.correia@grupocdrrj.com.br

Hospital São Lucas PUCRS, Porto Alegre, Rio Grande do Sul

- Principal Investigator: Giovani Gadonski, MD; ggadonski@yahoo.com.br
- Lead Coordinator: Adriana Conti, RN; adriana.conti@pucrs.br

Clínica de Doenças Renais - São Lourenço, Rio de Janeiro, Rio de Janeiro

- Principal Investigator: Inah Pecly, MD; inahpecly@terra.com.br
- Lead Coordinator: Camille Souza Paixão, RN; camille.souza@grupocdrrj.com.br

Fundação Pró Rim, Joinville, Santa Catarina

- Principal Investigator: Viviane Calice-Silva, MD, PhD; viviane.silva@prorim.org.br
- Lead Coordinator: Simone Ribeiro, RN; simoneprorim@gmail.com

Instituto Médico Nefrológico, Belo Horizonte, Minas Gerais

- Principal Investigator: Lizia Regina Ribeiro Caldeira, MD; lizia.caldeira@imnbh.com.br
- Lead Coordinator: Adailto Santos, RN; adailto.santos@imnbh.com.br

CETENE, São Paulo, São Paulo

- Principal Investigator: Rosilene Motta Elias, MD; rosilene.coelho@grupocetene.com.br

- Lead Coordinator: Andreia Barbosa Dos Santos, RN; andreia.santos@grupocetene.com.br

Hospital Alemão Oswaldo Cruz, São Paulo, São Paulo

- Principal Investigator: Américo Lourenço Cuvello-Neto, MD; cuvelloneto@gmail.com
- Lead Coordinator: Amanda Monteiro Virolli, RN; avirolli@haoc.com.br
